# Combining radiomics and molecular biomarkers: a novel economic tool to improve diagnostic ability in papillary thyroid cancer

**DOI:** 10.3389/fendo.2024.1378360

**Published:** 2024-08-14

**Authors:** Qingxuan Wang, Linghui Dai, Sisi Lin, Shuwei Zhang, Jing Wen, Endong Chen, Quan Li, Jie You, Jinmiao Qu, Chunjue Ni, Yefeng Cai

**Affiliations:** ^1^ Department of Thyroid Surgery, National Key Clinical Specialty (General Surgery), The First Affiliated Hospital of Wenzhou Medical University, Wenzhou, Zhejiang, China; ^2^ Zhejiang Key Laboratory of Intelligent Cancer Biomarker Discovery and Translation, First Affiliated Hospital, Wenzhou Medical University, Wenzhou, China; ^3^ Division of thyroid Surgery, Department of General Surgery, Nanjing Drum Tower Hospital, Affiliated Hospital of Medical School, Nanjing University, Nanjing, Jiangsu, China; ^4^ Department of Operating Room, The First Affiliated Hospital of Wenzhou Medical University, Wenzhou, Zhejiang, China; ^5^ Department of Breast Surgery, The First Affiliated Hospital of Wenzhou Medical University, Wenzhou, Zhejiang, China; ^6^ Department of Anesthesiology, The First Affiliated Hospital of Wenzhou Medical University, Wenzhou, Zhejiang, China; ^7^ Zhejiang Provincial Clinical Research Center for Malignant Tumor, Hangzhou, Zhejiang, China

**Keywords:** thyroid nodules, genetic test, PTC, radiomics, diagnostic model

## Abstract

**Background:**

A preoperative diagnosis to distinguish malignant from benign thyroid nodules accurately and sensitively is urgently important. However, existing clinical methods cannot solve this problem satisfactorily. The aim of this study is to establish a simple, economic approach for preoperative diagnosis in eastern population.

**Methods:**

Our retrospective study included 86 patients with papillary thyroid cancer and 29 benign cases. The ITK-SNAP software was used to draw the outline of the area of interest (ROI), and Ultrosomics was used to extract radiomic features. Whole-transcriptome sequencing and bioinformatic analysis were used to identify candidate genes for thyroid nodule diagnosis. RT-qPCR was used to evaluate the expression levels of candidate genes. SVM diagnostic model was established based on the METLAB 2022 platform and LibSVM 3.2 language package.

**Results:**

The radiomic model was first established. The accuracy is 73.0%, the sensitivity is 86.1%, the specificity is 17.6%, the PPV is 81.6%, and the NPV is 23.1%. Then, CLDN10, HMGA2, and LAMB3 were finally screened for model building. All three genes showed significant differential expressions between papillary thyroid cancer and normal tissue both in our cohort and TCGA cohort. The molecular model was established based on these genetic data and partial clinical information. The accuracy is 85.9%, the sensitivity is 86.1%, the specificity is 84.6%, the PPV is 96.9%, and the NPV is 52.4%. Considering that the above two models are not very effective, We integrated and optimized the two models to construct the final diagnostic model (C-thyroid model). In the training set, the accuracy is 96.7%, the sensitivity is 100%, the specificity is 93.8%, the PPV is 93.3%, and the NPV is 100%. In the validation set, the accuracy is 97.6%, the sensitivity remains 100%, the specificity is 84.6%, the PPV is 97.3%, and the NPV is 100%.

**Discussion:**

A diagnostic panel is successfully established for eastern population through a simple, economic approach using only four genes and clinical data.

## 1 Introduction

Thyroid cancer is the most increased endocrine malignant tumor in recent years (1). Papillary thyroid cancer (PTC), the most common pathological type, accounts for 90% of all thyroid cancers (1). Because of the enormous discrepancies in treatments, a preoperative diagnosis to distinguish benign from malignant thyroid nodules accurately and sensitively is urgently important. An accurate preoperative diagnosis may efficiently assist patients with thyroid cancer in their treatment and help patients with benign nodule avoid unnecessary thyroid surgery, which helps prevent the corresponding surgical complications, such as injury of recurrent laryngeal nerve and hypoparathyroidism.

Fine-needle aspiration (FNA) is the most frequently recommended tool in thyroid preoperative diagnosis (2). The accuracy of FNA is about 72.8% to 87.2%, which is primarily correlated with histopathology (3, 4). However, its limitations include the need for a highly experienced cytopathologist for accurate interpretation and the frequently indeterminate cytology. About 17% (10%–26%) of FNAB were reported as indeterminate, and 6% (1%–11%) were nondiagnostic, while 72% median (range 62%–85%) of undertaken FNAB were benign, and 5% (1%–8%) were malignant (5). Consequently, patients with uncertain cytological results need to receive repeated FNA or invasive diagnostic surgeries, which increases the potential risks of relevant postoperative complications and unnecessary surgical intervention.

In recent years, molecular detection has become a promising diagnostic tool for physicians to distinguish benign from malignant thyroid nodules (6). A microarray-based test, combining the expression levels of 167 genes (Afirma^®^ Gene Expression Classifier) can diagnose thyroid nodules with 92% sensitivity and 52% specificity (7). Another classifier, named ThyroSeq v3, a DNA- and RNA-based next-generation sequencing assay, can achieve accurate diagnosis with 98% sensitivity, 81.8% specificity, and 90.9% accuracy by using next-generation sequence (8). Other panels, such as RosettaGX and ThyGenX/ThyraMIR, also show good prospects (9).

Despite these contributions to the development of molecular diagnosis, these approaches have limitations. First, the high panel costs limit their clinical applications, especially for undeveloped and less developed countries. Second, the current research objects are often confined to European and American patients, while similar research on Asian patients are rare. Due to the genetic differences between different populations, in-depth research on molecular diagnosis applied to Asian patients is necessary.

Previous studies have shown that ultrasound features of thyroid tumors, including tumor size, capsule invasion, and microcalcification, are independent predictors of LN metastasis in patients with PTC and have been widely used to screen for thyroid nodules (10). However, a high level of clinician experience is required, and visual examination has limitations. Radiomics is an emerging technology that transforms medical images into mineable data by extracting a large number of quantitative features from digital images (11, 12). Radiomic analysis epitomizes the pursuit of precision medicine, which emphasizes providing the right treatment to the right patient at the right time (13). Currently, radiomic analysis has been applied to a variety of diseases, for instance, by Haralick texture analysis and mining apparent diffusion coefficient and MR image information, it helps build panels to differentiate between cancerous and noncancerous prostate tissues (14).

Our study combines molecular markers and radiomics to establish an economical, effective model for the preoperative diagnosis of thyroid nodules based on the Chinese population.

## 2 Method

### 2.1 Patients and tissue collection

The research protocol was approved by the Ethics Committee of the First Affiliated Hospital of Wenzhou Medical University. The Primary PTC samples or benign thyroid lesions including samples of their healthy thyroid tissue in neighboring areas used in the research are from patients undergoing primary surgeries in the First Affiliated Hospital of Wenzhou Medical University. 19 pairs of PTC samples (19 PTC tumor samples and 19 paired matched adjacent normal thyroid tissue samples) were collected from June 2011 to August 2013. Another 115 samples (86 PTC samples and 29 benign samples) for training set and validation set were collected from December 2013 to December 2016. The inclusion criteria are as follows: surgical indications on the patient; the tumor size larger than 5 mm; a diagnosis of papillary thyroid cancer or benign thyroid lesions; a clear postoperative pathological diagnosis; a sharp ultrasonoscope image and the patient’s approvement. The exclusion criteria are as follows: the tumor with a ≤5mm size; an ambiguous postoperative pathological diagnosis; the existence of other malignant tumors; surgical contraindications on the patient; and an unsharp ultrasonoscope image.

The removed sample in the surgery was quickly frozen in liquid nitrogen and stored in a refrigerator with a temperature of -80°C, while the sample sets used in the training and verification of this research are all quick-frozen. The retrospective review of histopathological slides in each case was carried out by three pathologists with a minimum of 5 years’ experience to verify the histological diagnosis and ensure the sufficient cancer in the tumor. All the patients have signed the informed consent, allowing scientific utilizations of their biological materials and information within the law permission.

### 2.2 Next generation sequencing

ated using TRIzol reagent according to manufacturer’s protocol (Life Technologies, Carlsbad, CA). And after a quality test, we used Ion Total RNA-Seq Kit v2.0 (Life Technologies, Carlsbad, CA) to prepare the cDNA libraries according to the manufacturer’s instructions. Then, the cDNA libraries were sequenced by using Illumina Hiseq 2500 according to the commercially available instructions. Genes satisfying the following conditions can be filtered out with the Ebseq algorithm: 1) fold change (FC) > 2, for upward or downward adjustment;2) fate discovery rate (FDR) < 0.05 and 3) P-Value < 0.05.

### 2.3 RNA isolation and RT-qPCR

Following the manufacturer’s instruction, TRIZOL (Invitrogen)is used to segregate and reverse transcript total RNA (TOYOBO, Japan). Subsequently, THUNDERBIRD SYBR qPCR Mix (TOYOBO, Japan) is employed to carry out three quantitative real-time PCR (RT-qPCR) analyses on ABI prism 7500 sequence detection system. According to GAPDH mRNA level, the primer sequence is as follows: CLDN10:5’- GAGGCTCCGATAAAGCCAAAG-3’(forward)and 5’-ACAGAGCGGCTCCTAATTCAT-3’ (reverse), HMGA2: 5’-ACCCAGGGGAAGACCCAAA-3’(forward) and 5’-CCTCTTGGCCGTTTTTCTCCA-3’(reverse), LAMB3:5’-GCAGCCTCACAACTACTACAG-3’(forward) and 5’-CCAGGTCTTACCGAAGTCTGA-3’ (reverse);GAPDH: 5’-GGTCGGAGTCAACGGATTTG-3’(forward) and 5’-ATGAGCCCCAGCCTTCTCCAT-3’(reverse). BRAF V600E mutation is detected by ARMS-PCR (amplification refractory mutation system) (15). When Ct value ≥ 38, it is recognized as wild type. When Ct value < 38, we further calculate the ΔCt value. When ΔCt < 9, it is recognized as mutant type, otherwise, it is wild type.

### 2.4 Ultrasound examination

Preoperative ultrasound examinations are carried out on all patients included. The ultrasound examinations are performed using a GE LOGIQ E9 ultrasound system with a 6–15L linear array probe set at 11–13 MHz. During the examination, each patient is placed in a supine position on the examination bed, with the neck fully extended, and the patient is allowed to breathe calmly to examine the thyroid gland and its surrounding tissues. The number and size of nodules in the thyroid gland, their borders, internal structure, internal echogenicity, and the degree of calcification are recorded and the TI-RADS grade was assessed. Finally, thyroid nodules are recorded and reported.

### 2.5 Radiomics analysis

The ultrasound images are exported from the imaging system, being converted from jpg format to medical digital imaging and communication format. Subsequently, the ITK-SNAP software (http://www.itksnap.org/pmwiki/pmwiki.php) is used to draw the outline of the area of interest (ROI), which is carried out by three ultrasound radiologists with a minimum experience of five years. Then, according to the manufacturer’s instruction, the nodules were analyzed with Ultrosomics (GE healthcare, version 1.1) to extract radiomic features and construct and evaluate models that enables a prediction of benign and malignant thyroid nodules based on the expression levels of four genes.

### 2.6 Statistical analysis

Statistical analysis is performed using SPSS 25.0 (SPSS Inc., Chicago, IL, USA). Normally distributed data (Data complies with normal distribution) are shown as mean ± standard variation (SD) and compared to the testing, and chi-square analysis or Fisher’s exact test as appropriate. P values meeting following conditions are considered statistically significant: 1) All p values were two sided, 2) p values < 0.05. Finally, values including sensitivity, specificity, positive predictive value (PPV), negative predictive value (NPV) and accuracy are calculated to assess the diagnostic performance of these tests.

## 3 Results

### 3.1 Flowchart of the study

**Figure 1:**
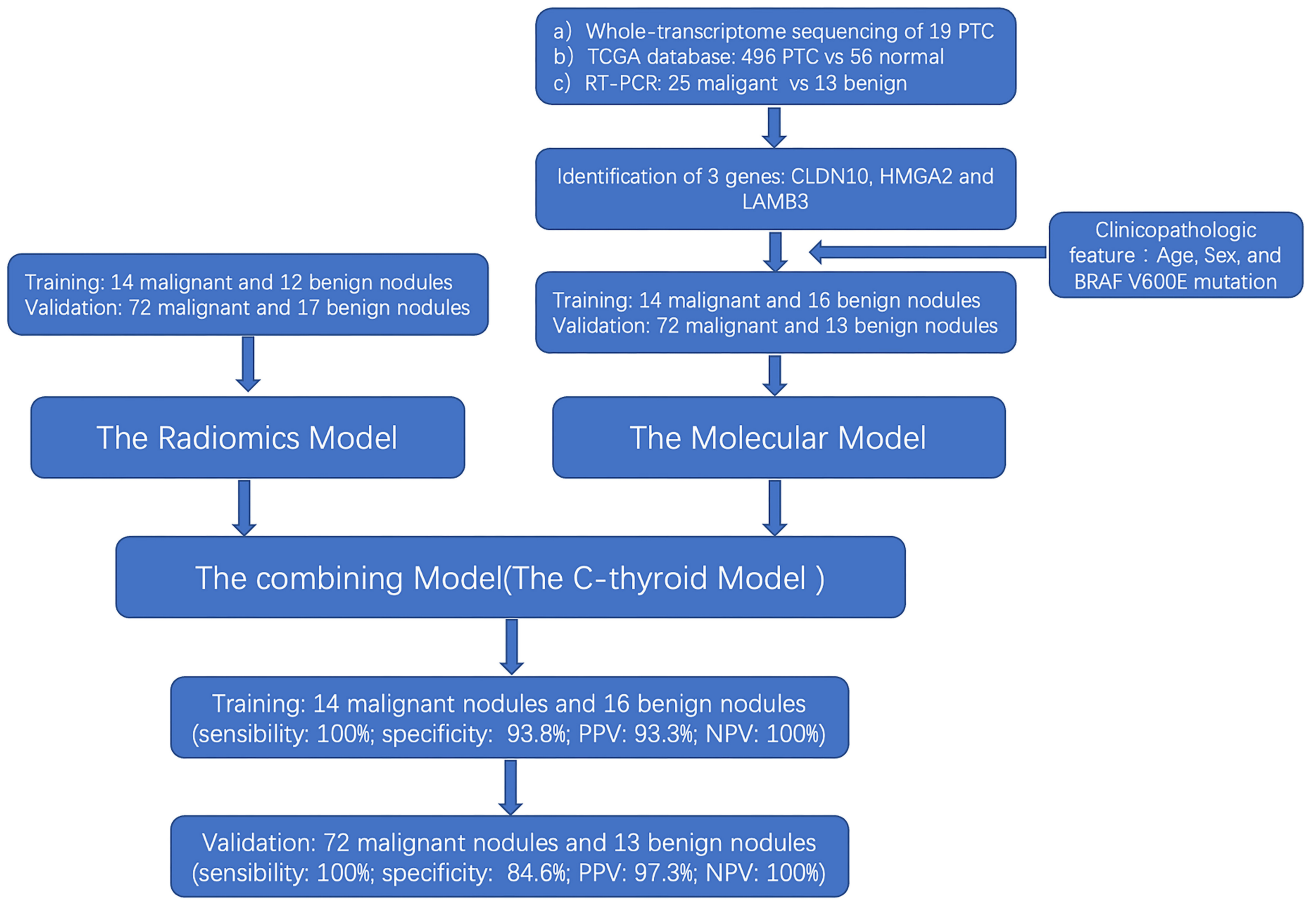
The flowchart of the study.

The flow chart of our study design is shown in **Figure 1**. A total of 115 patients were identified in the training set (14 malignant nodules and 12 benign nodules) and the validation set (72 malignant nodules and 17 benign nodules) to establish a radiomic model to distinguish the thyroid nodules. Then, training set (14 malignant nodules and 16 benign nodules) and validation set (72 malignant nodules and 13 benign nodules) were used to establish a molecular model. Considering that the above two models are not very effective, we integrated and optimized the two models to construct the final diagnostic model (C-thyroid model). 30 patients were in the training set (14 malignant nodules and 16 benign nodules), and 85 patients (72 malignant nodules and 13 benign nodules) were in the validation set. Baseline data of 115 cases was shown in **Supplementary Table 1**.

### 3.2 Establishment of the radiomic model

Patients were randomly divided into the training group and the validation set. Data from the training set were used to screen features and construct radiomic model, and the validation set was used for validation. Multiple feature selection was performed to identify reliable features and reduce redundancies. Based on the radiomic features selected from the training set, six algorithms were used in to construct radiomic models for prediction: KNN, LR, DT, linear-support vector machine, Gaussian-SVM, and polynomial-SVM. Finally, the accuracy of the radiomic models was determined and validated by calibration-decision-curve analysis and AUC. The sensitivity is 86.1%, the specificity is 17.6%, the PPV is 81.6%, and the NPV is 23.1% (**Table 1**).

**Table 1:** The performance of the Radiomics Model.

	The Radiomics Model			Predictive value	
	Positive	Negative	Total	Sensitivity	86.1%
Malignant	62	14	76	Specificity	17.6%
Benign	10	3	13	PPV	81.6%
Total	72	17	89	NPV	23.1%
				Accuracy	73.0%

PPV, positive predictive value; NPV, negative predictive value.

### 3.3 Establishment of the molecular model

To filter for differentially expressed genes, whole-transcriptome sequencing was performed on 19 pairs of PTC samples. Total RNA was extracted from tissue samples using TRI-ZOL Reagent (Invitrogen) according to the manufacturer’s protocol. After the quality test, the cDNA libraries were prepared using Ion Total RNA-Seq Kit v2.0 (Life Technologies) according to the manufacturer’s instructions. The cDNA libraries were then processed for sequencing using Illumina Hiseq 2500 according to the commercially available protocols. Before reads mapping, clean reads were obtained from the raw reads by removing the adaptor sequences, reads with > 5% ambiguous bases, and low- quality reads. The clean reads were then aligned to the human genome (version: GRCH37) using the MapSplice program (v2.1.6, University of Kentucky, Lexington, KY, USA). We applied Ebseq algorithm to screen out the differently expressed genes.

We identified a total of 212 differentially expressed genes from the 19 paired-PTC tissue samples (log_2_FC > 1.0, FDR < 0.05 and P-Value < 0.05). When the filtering conditions are further improved, only 39 differentially expressed genes were identified (log_2_FC > 5.0, T counts > 1000, and P value < 0.01). By combining the TCGA database (Log_2_FC > 1, T (FPKM) > 3, N (FPKM) < 2), 26 genes that are stably highly expressed in both our center and the TCGA were selected. Furthermore, by combining GEO data (GSE33630, GSE29265, GSE27155), 11 genes that are stably highly expressed were identified. Subsequently, CLDN10, HMGA2, and LAMB3 were ultimately identified through RT-qPCR validation. Then, the expression of these three over expressed genes in 19 paired PTC samples (19 PTCs and 19 NTs) were investigated using sequencing data.

**Figure 2:**
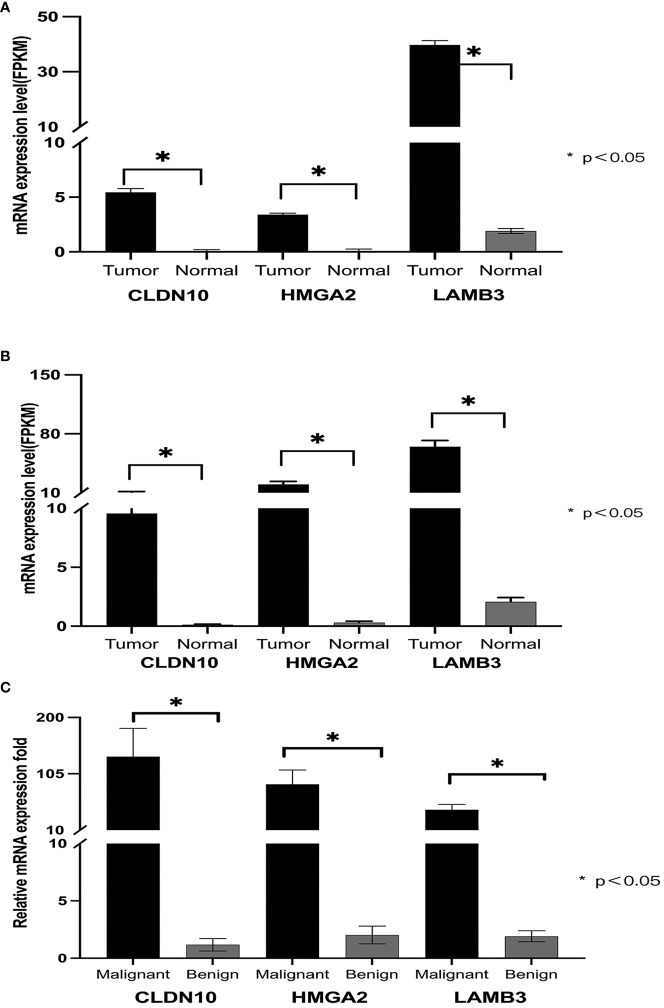
Expression of CLDN10, HMGA2, and LAMB3 genes in PTC. **(A)** shows that in the TCGA database, the mRNA expression levels of CLDN10, HMGA2, and LAMB3 genes in papillary thyroid cancer were significantly higher than those in normal tissue. **(B)**, among the 19 transcriptome sequencing data from our center, the mRNA expression levels of CLDN10, HMGA2, and LAMB3 genes were significantly higher in papillary thyroid cancer than in normal tissues. **(C)**, the mRNA expression levels of CLDN10, HMGA2, and LAMB3 genes were significantly higher in malignant puncture specimens than in benign puncture specimens.

All three genes showed significant differential expressions between PTC and normal tissue (**Figure 2B**). For a further confirmation of the relationship between PTC and the genes CLDN10, HMGA2, and LAMB3, the mRNA expression of these three genes in 496 PTC and 56 noncancerous cases in the TCGA database was analyzed. Based on the TCGA database, the patients were divided into two groups (Tumor group and Normal group), of which 56 cases were normal controls, and high expressions were also observed on CLDN10, HMGA2, and LAMB3 (**Figure 2A**). The highly expressed CLDN10, HMGA2, and LAMB3 were also validated with 38 FNBA samples (25 malignant and 13 benign, **Figure 2C**). The BRAF gene also possesses an excellent ability to distinguish between benign and malignant tumors (15–18). A diagnostic model combining both BRAF V600E mutation and BSRTC (Bethesda System for Reporting Thyroid Cytopathology) grading showed the highest sensitivity of 82.9% and specificity of 85.4% (15). Another study found that, the sensitivity and specificity of the BRAF V600E mutation test alone for PTC diagnosis were 76.71% and 100.0%, respectively, which increased to 96.62% and 88.03%, respectively, when combining the BRAF V600E mutation test with cytology (16). A systematic review and meta-analysis showed that the overall sensitivity and specificity for BRAF V600E for the diagnosis of thyroid malignancy was 0.40 (95% CI: 0.32–0.48) and 1.00 (95% CI: 0.98–1.00) respectively (18). Therefore, the BRAF gene was also included in our study. No BRAF V600E mutation was detected in all 29 benign nodules in our study. Among all 86 malignant nodules, BRAF V600E mutation was detected in 73 cases (**Table 2**).

**Table 2:** BRAF status of 115 enrolled cases.

	BRAF V600E mutant type	BRAF V600E wild type	
Malignant	73	13	86
Benign	0	29	29
	73	42	

Finally, four genes, namely, CLDN10, HMGA2, LAMB3, and BRAF, were selected for the molecular model construction (absolute mRNA expression for CLDN10, HMGA2, LAMB3, and the status of BRAF-V600E mutation). The accuracy is 85.9%, the sensitivity is 86.1%, the specificity is 84.6%, the PPV is 96.9%, and the NPV is 52.4% (**Table 3**).

**Table 3:** The performance of the Molecular Model.

	The Molecular Model			Predictive value	
	Positive	Negative	Total	Sensitivity	86.1%
Malignant	62	2	64	Specificity	84.6%
Benign	10	11	21	PPV	96.9%
Total	72	13	85	NPV	52.4%
				Accuracy	85.9%

PPV, positive predictive value; NPV, negative predictive value.

## 4 Establishment of combining model (C-thyroid model)

### 4.1 SVM model

**Figure 3:**
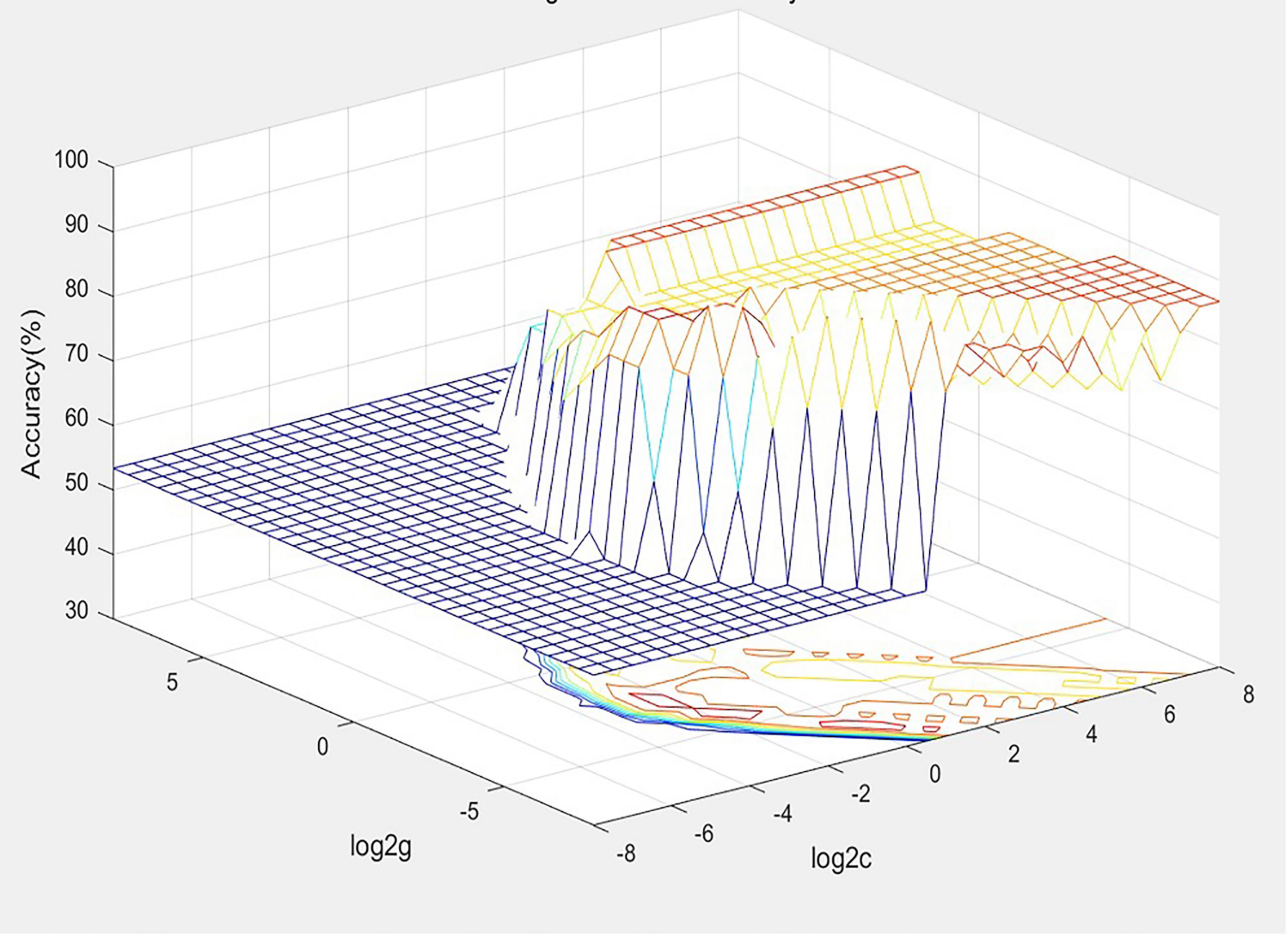
The SVM model of diagnostic panel.

With the acquisition of radiomic data and the assignment of variables, a unified database was established after the assignment of 30 training set variables, and the best SVM diagnostic model for the diagnosis of thyroid nodules was established based on the METLAB 2022 platform and LibSVM 3.2 language package (**Figure 3**).

The model was debugged using C-SVC, RBF kernel functions, and the grid search method, with the grid c bound, grid c step, grid g bound, and grid c step set as −8 to 8, 0.5, −8 to 8, and 0.5, respectively, and the multiplier for cross-validation set as 5. Patients were divided into two groups, with positive values indicating malignant, and negative values indicating benign.


Plabel=sgn(Σni = 0 wi exp(−gamma|(xi−x)|2+b))

### 4.2 Training set: building a combining panel

Based on the training set, a diagnostic panel (C-thyroid model) combining four genes (BRAF, CLDN10, HMGA2, and LAMB3) and radiomic models was successfully established using the SVM model. The results showed the panel has a good discriminatory ability with 96.7% of the thyroid nodule samples correctly classified. The sensitivity is 100%, the specificity is 93.8%, the PPV is 93.3%, and the NPV is 100%.

### 4.3 Validation set: a validation of panel in external independent samples

**Figure 4:**
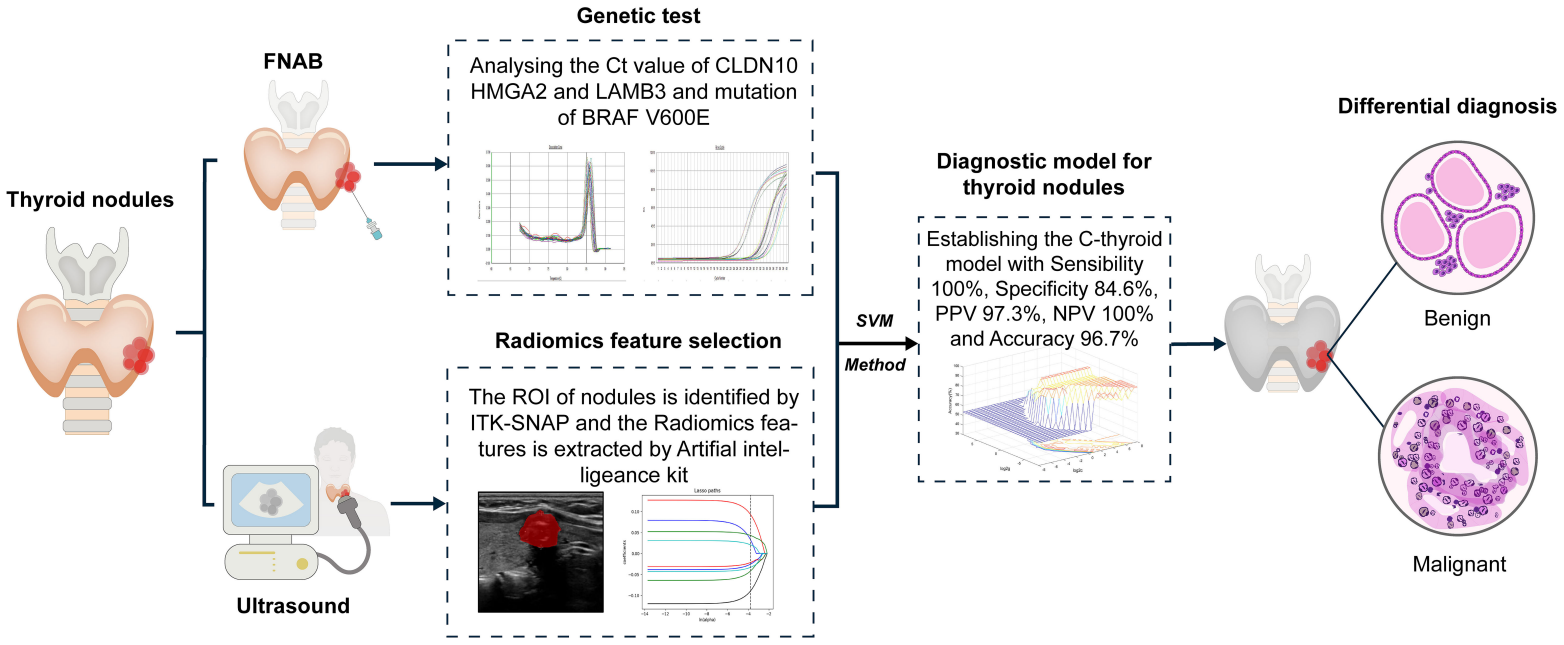
Clinical application process of the diagnostic model.

To validate the performance of C-thyroid model, the mRNA expression levels of the three genes were measured using RT-qPCR in 85 additional samples. Similar to the previous data, the relative expression levels of the three genes were significantly upregulated. The panel also achieved a high accuracy of 97.6% on the validation set. The sensitivity remains 100%, the specificity is 84.6%, the PPV is 97.3%, and the NPV is 100% (**Table 4**). To verify the effectiveness of C-thyroid model in indeterminate nodules, we also tested the model on 10 FNA samples with indeterminate nodules, correctly identifying 9 out of 10 with 90% accuracy (**Supplementary Table 2**). The results showed the performance of the C-thyroid model is satisfactory. The C-thyroid model analysis pipeline is depicted in **Figure 4**.

**Table 4:** The performance of the C-thyroid Model.

	The C-thyroid Model			Predictive value	
	Positive	Negative	Total	Sensitivity	100%
Malignant	72	2	74	Specificity	84.6%
Benign	0	11	11	PPV	97.3%
Total	72	13	85	NPV	100%
				Accuracy	97.6%

PPV, positive predictive value; NPV, negative predictive value.

## 5 Discussion

In recent years, thyroid cancer, especially PTC, has become the most common endocrine tumor (19). With the development of medical laboratory techniques, in daily clinical practices, physicians encounter an increasing number of patients with thyroid nodules found accidentally in imaging examinations, most of which are benign (8). Accurately distinguishing benign from malignant nodules is important so that patients with cancer can receive timely, appropriate treatment, while patients with benign nodules could avoid unnecessary surgery, which often brings them a high incidence of associated postoperative complications.

Although ultrasound is the clinically recommended method for the detection of thyroid nodules as the first choice, it has certain limitations (20). Ultrasonic diagnosis is limited by the clinical experience of the imaging physician, which is a subjective diagnosis. FNA is strongly recommended by the guidelines. However, FNA is occasionally limited by the frequently indeterminate cytology. Traditional pathological diagnosis and ultrasound accuracy are limited by the imaging physician’s clinical work experience, which is a subjective diagnosis. By contrast, radiomic analysis, as an extension of accurate medicine in the 21st century, is of great value in medical diagnosis and prognosis. In contrast to the traditional approach of treating medical images solely for visual interpretation, medical images, from which a high-throughput extraction of quantitative features is carried out, are transformed into mineable data and then analyzed to support decision making. Studies with practical results are already available. For example, Simon K B Spohn et al. reported the feasibility of imaging histology in distinguishing cancerous and noncancerous prostate tissue and its value in risk stratification assessment (21). Shan Tan et al.’s findings showed the Spatial-FDG PET feature could be a useful predictor of pathologic tumor response to neoadjuvant chemoradiotherapy for esophageal cancer (22). These studies indicate, in varying degrees, the possibility of imaging histology application in determining the benignity or malignancy of thyroid nodules. However, the clinical application of imaging histology is limited by economic factors because the price of commercially available panels covering imaging histology analysis techniques is generally high.

Facing this challenge, several diagnostic panels have been clinically reported using high-throughput technologies, such as next-generation sequences and microarray (8, 23), which are currently approved for clinical trials. A microarray-based test, combining the expression levels of 167 genes (Afirma^®^ Gene Expression Classifier) can diagnose thyroid nodules with 92% sensitivity and 52% specificity (7). Another classifier, named ThyroSeq v3, a DNA- and RNA-based next-generation sequencing assay, achieves accurate diagnosis with 98% sensitivity, 81.8% specificity, and 90.9% accuracy by using next-generation sequence (8). Related studies show positive progress (24, 25). However, its clinical application in less developed regions and countries is still limited due to the high cost. Most of the trials reported so far have been conducted on patients with thyroid nodules from Western countries. These panels have not been proven feasible in Asian populations. Many studies have shown that Asians and Westerners have different genetic backgrounds. For instance, Xing M et al. reported BRAF mutation occurred in about 45% of patients with PTC in the USA (26), whereas the rate reported in Asians was much higher, namely, 63.7% in the study from Jin L et al. (27) and 58% in the study from Lee JH (28). Given the genetic differences in thyroid nodules between Asians and Europeans, a diagnostic panel for Asians is urgently necessary.

In our study, we first established a radiomic model. However, the effect is not satisfactory. Then, we identified three genes through whole-transcriptome sequencing and bioinformatic analysis, named CLDN10, HMGA2, and LAMB3. Based on these genes, a molecular model was established. The effect has been significantly improved, but it is still not ideal.

The three genes identified in our study are highly consistent with another study from South America (29). This indicates that the expression differences of these three genes are relatively small in different populations. These three genes may be ideal diagnostic markers generally in PTC. This requires further in-depth research.

In order to establish a more ideal model, we integrated and optimized the two models to construct the final diagnostic model (C-thyroid model). The performance was further validated by independent external samples, in which the sensitivity is 100%, the specificity is 84.6%, the PPV is 97.3%, and the NPV is 100%. Our preliminary research also confirmed that the model still maintains good accuracy in 10 indeterminate nodules. Considering the small sample size, further research needs to be carried out. This is the first panel to combine radiomic analysis and molecular test, which is highly innovative. Furthermore, our panel is easy to operate without professional training. Finally, our panel has great economic advantages in reducing financial burdens of patients.

Our paper has limitations. First, this is a retrospective design because ultrasonography is a part of patients’ diagnostic examination, and imaging histology is applied retrospectively to the available data. Second, the sample size of this study is confined to patients from a single hospital; hence, large-scale, multicenter clinical studies are necessary for further validations. Third, long-term follow-up data are missing, which weakens the study’s conclusion dynamics.

## 6 Conclusion

In this paper, a diagnostic panel is successfully established through a simple, economic approach using only four genes and imaging data that have performed well in Chinese patients. It is the first panel to combine radiomic analysis and molecular test. It may contribute to the foundation for a powerful, practical, and economic molecular diagnostic tool for clinical use in the future.

## Data Availability

The original contributions presented in the study are included in the article/**Supplementary Material**, further inquiries can be directed to the corresponding author/s.
