# Supplementary material for: Combining radiomics and molecular biomarkers: a novel economic tool to improve diagnostic ability in papillary thyroid cancer
Source: Front Endocrinol (Lausanne). 2024 Aug 14;15:1378360. doi: 10.3389/fendo.2024.1378360 (PMC11349561; doi:10.3389/fendo.2024.1378360)
Supplement: Supplementary file 3 [file Table1.docx]

| Supplementary Table 1. Baseline data of PTC (n=86) and benign thyroid nodules (n=29) | | |
| --- | --- | --- |
| Characteristics | PTC（n=86） | benign（n=29） |
| Age at diagnosis, y |  |  |
| Mean±SD | 41.05±12.76 | 48.48±10.23 |
| < 45 y | 52 | 14 |
| ≥ 45 y | 34 | 15 |
| Gender |  |  |
| Female | 28 | 7 |
| Male | 58 | 22 |
| Tumor size in mm |  |  |
| Mean±SD | 16.26±7.21 | 31.59±14.95 |
| ≤ 10mm | 20 | 3 |
| > 10mm | 66 | 26 |
| Hashimoto’s thyroiditis |  |  |
| Yes | 39 | 7 |
| No | 47 | 22 |
| Lymph node metastasis |  |  |
| Yes | 62 | NA |
| No | 24 | NA |
