# Supplementary material for: Combining radiomics and molecular biomarkers: a novel economic tool to improve diagnostic ability in papillary thyroid cancer
Source: Front Endocrinol (Lausanne). 2024 Aug 14;15:1378360. doi: 10.3389/fendo.2024.1378360 (PMC11349561; doi:10.3389/fendo.2024.1378360)
Supplement: Supplementary file 4 [file Table2.docx]

| Supplementary Table 2. Results of 10 FNA samples with indeterminate nodules | | | |
| --- | --- | --- | --- |
| Patient ID | Bethesda | Pathological result | C-thyroid model result |
| 001 | III | nodular goiter | benign |
| 002 | IV | follicular adenoma | benign |
| 003 | V | PTC | malignant |
| 004 | III | nodular goiter | benign |
| 005 | V | PTC | malignant |
| 006 | III | nodular goiter | benign |
| 007 | III | PTC | malignant |
| 008 | IV | PTC | benign |
| 009 | III | nodular goiter | benign |
| 010 | V | PTC | malignant |
